# Valorization of fermented orange pulp as a sustainable feed ingredient: Impacts on broiler growth, immune system, meat quality and lipid oxidation

**DOI:** 10.1016/j.psj.2025.105964

**Published:** 2025-10-09

**Authors:** Michael Goliomytis, Panagiotis Simitzis, Agori Karageorgou, Nicoleta Michalea, Kyriaki Belesi, Maria-Eleni Mougiou, Vasiliki Syritou, Ariadne-Loukia Hager-Theodorides, Ioannis Stavrakakis, Spyridon Ntougias

**Affiliations:** a Laboratory of Animal Breeding and Husbandry, Department of Animal Science, Agricultural University of Athens, 75 Iera Odos, 11855 Athens, Greece; b Laboratory of Wastewater Management and Treatment Technologies, Department of Environmental Engineering, Democritus University of Thrace, 12 Vas. Sofias, 67132 Xanthi, Greece

**Keywords:** Fermented orange pulp, Broiler, Meat oxidative stability, Microbial inocula, Cholesterol

## Abstract

The citrus industry produces vast quantities of by-products, such as orange pulp (**OP**), which can be valorized as alternative feed ingredients within sustainable poultry production systems. Fermentation is a promising biotechnological approach that enhances the nutritive and functional properties of agro-industrial by-products such as OP. In this study the effects of dietary inclusion of OP, either unfermented or fermented for 20 days, on broiler performance, carcass traits, meat quality, oxidative stability, immune parameters and serum lipid profiles were investigated. A total of 176 Cobb 500 broilers were assigned to four dietary treatments: control (basal diet), unfermented OP, OP inoculated with mixed inoculum without fermentation (**T0**), and OP inoculated with mixed inoculum and fermented for 20 days (**T20**), all at 7 % inclusion. Growth performance and most of carcass characteristics were not significantly affected by OP supplementation (*P* > 0.05). However, dietary OP influenced skin and meat coloration, with unfermented OP groups showing increased redness in comparison with either C or T20 groups. Fermentation significantly reduced malondialdehyde (**MDA**) levels in breast meat during storage, in comparison with groups fed with non-fermented OP (*P* < 0.05), thus showing improved oxidative stability. Serum total cholesterol was reduced in all OP-fed groups but at the same time high density lipoprotein (**HDL**) tended to increase (*P* < 0.1). A subtle immunosuppression was detected in broilers fed with unfermented OP in comparison with controls as shown from the reduced number of T cytotoxic cells determined in serum of OP and T0 groups (*P* < 0.05). These results support the inclusion of OP, particularly fermented, as a sustainable feedstuff that enhances meat quality and lipid profile without impairing broiler growth. Valorization of such an agro-industrial waste enhances circularity and promotes sustainable poultry feeding with respect to environmental protection.

## Introduction

Significant amounts of orange pulp (**OP**), consisting of peels, pulp, seeds, and membranes, are generated globally, accounting for approximately 50 % of the fresh fruit weight (Mamma and Christakopoulos, 2014; FAO, 2021). Due to their high moisture and carbohydrate content, these residues of the citrus industry are often treated as waste and can favor microbial spoilage and increase the economic cost for their treatment. However, these agro-industrial by-products can be considered as alternative feed ingredients and, within the framework of a circular economy, may promote sustainable animal nutrition (Georganas et al., 2023).

Orange pulp is a by-product abundantly found worldwide with considerable nutritional value due to the high level of carbohydrates, including pectin and bioactive compounds like flavonoids and phenolic acids, which exert antioxidant and health-promoting effects when fed to animals (Tripodo et al., 2004; Bampidis and Robinson, 2006). Despite these benefits, the direct inclusion of OP in poultry diets may be disadvantageous due to the high fiber content, especially the NDF/ADF fraction, the presence of anti-nutritional compounds, and variable palatability, which can reduce feed efficiency and growth performance (Murao et al., 2008; Goliomytis et al., 2018).

Fermentation is a low-cost biotechnological approach that enhances the nutritive and functional properties of agro-industrial by-products, including OP. A range of biomolecules like complex carbohydrates and anti-nutritional factors, can be degraded by various fermentative microorganisms, like Aspergillus or Lactobacillus, resulting in improved nutrient availability and the production of a fermentation substrate enriched in beneficial microbial metabolites (Olukomaiya et al., 2019; Faria et al., 2023) that exert significant antimicrobial activity (Kang et al., 2017). Moreover, antioxidant activity and bioaccessibility of phenolic compounds may be enhanced through fermentation of citrus residues, resulting in a high value-added product rich in organic acids and exopolysaccharides, with possible beneficial properties for animal health and oxidative stability of meat products (Saeed et al., 2024; Yu et al., 2025). Thus, fermentation of orange pulp can be considered as a sustainable treatment method for citrus waste valorization, resulting in a microbially-enriched feedstuff with potential benefits for poultry production and meat quality.

There are several studies evaluating the effects of orange by-products in pigs (Cerisuelo et al., 2010; Belloumi et al., 2023), steers (Luzardo et al., 2021), dairy cattle (Williams et al., 2018; Martins et al., 2021), dairy ewes (Fegeros et al., 1995), lambs (Lanza et al., 2015) and broiler chickens (Abbasi et al., 2015; Diaz-Vargas et al., 2019; Zoidis et al., 2022). However, only one study addresses the effects of dietary fermented OP on broilers (Oluremi et al., 2010) which focuses solely on performance effects. Considering the vulnerability of poultry meat to lipid oxidation which in turn affects shelf life, sensory attributes and consumer acceptance, the use of citrus residues, as feedstuff which can further be enhanced through solid state fermentation, could be a promising prospective.

Therefore, the aim of the present study was to investigate the effects of dietary inclusion of fermented OP on the growth performance, carcass traits, meat quality parameters, lipid oxidative stability, blood lipid profiles and immune parameters of broiler chickens. The outcomes of the present study aim to contribute to the sustainable employment of citrus juice industry by-products in broiler farming and to further assess whether fermentation can improve the nutritional benefits of OP as a feed ingredient.

## Material and methods

### Ethics approval

The methods used in the present experiment were approved by the Research Ethics Committee of the Agricultural University of Athens under protocol number 81/2023.

### Animals, housing, and experimental design

A total of 176 1-day-old Cobb 500 straight run broiler chickens, obtained from a commercial hatchery, were reared in the experimental station of the Agricultural University of Athens under a controlled environment. Birds were allocated to four treatment groups: control (C), fed with a basal diet; O, fed with orange pulp (OP) at 70 g/kg feed; T0 fed with OP in the presence of mixed inoculum without fermentation at 70 g/kg feed and T20, fed with OP fermented with mixed inoculant for 20 days, at 70 g/kg feed. Starter diets (1-10 days) contained OP, fermented or not, at 50 g/kg feed for a gradual adaptation of chickens to OP consumption. All diets were in mash form, isocaloric and isonitrogenous. The level of 70 g of OP per kg of feed was chosen as the upper limit of OP supplementation for formulating diets that meet nutrient requirements of broilers. The birds were randomly allocated in 16 pens, in 4 replicate pens per treatment, each with an area of 1.5 m², until 42 days of age. Each pen housed 11 chickens. Upon arrival, each chick was individually weighed. The lighting schedule initially provided 23 hours of light and 1 hour of darkness (23L:1D), which was reduced to 18L:6D on day 7. This schedule remained constant until day 28, after which it gradually increased by one hour a week and back to 23L:1D at slaughter. Environmental conditions and management practices followed standard Cobb broiler guidelines (Cobb Broiler Management Guide, 2021). Feed and water were available for *ad libitum* consumption. The composition and chemical analysis of the three diets offered throughout the experimental period (starter, grower, finisher), along with chemical analysis of fermented and non-fermented OP used in diets, are presented in Table 1. Individual body weight (BW), feed intake (FI) per pen and feed conversion ratio (FCR) per pen were recorded weekly.

**Table 1:** Ingredients and chemical composition of the diets used (g/100 g).

		Starter, 1 to 10 days				Grower, 11 to 24 days				Finisher, 25 to 42 days					
Diets	C	OP	T0	T20	C	OP	T0	T20	C	OP	T0	T20	Orange Pulp^2^	T0 orange Pulp^2^	T20 orange Pulp^2^
Ingredients															
Maize	60.2	53.5	53.5	53.5	64.6	55.2	55.2	55.2	68	58.6	58.6	58.6			
Soybean meal 47 %	34	34	34	34	30	30	30	30	26.8	26.8	26.8	26.8			
Orange pulp	0	5	0	0	0	7	0	0	0	7	0	0			
T0 orange pulp	0	0	5	0	0	0	7	0	0	0	7	0			
T20 orange pulp	0	0	0	5	0	0	0	7	0	0	0	7			
Soybean oil	1	2.75	2.75	2.75	1.5	4	4	4	1.8	4.3	4.3	4.3			
Limestone	1	0.89	0.89	0.89	0.7	0.5	0.5	0.5	0.7	0.56	0.56	0.56			
Monocalcium phosphate	0.8	0.8	0.8	0.8	0.4	0.4	0.4	0.4	0.2	0.21	0.21	0.21			
Methionine	0.15	0.17	0.17	0.17	0.1	0.13	0.13	0.13	0	0.03	0.03	0.03			
Threonine	0.15	0.16	0.16	0.16	0.1	0.12	0.12	0.12	0	0.02	0.02	0.02			
Biolysine HCL	0.2	0.23	0.23	0.23	0.1	0.15	0.15	0.15	0	0.03	0.03	0.03			
Vitamin-trace-mineral premix^1^	2.5	2.5	2.5	2.5	2.5	2.5	2.5	2.5	2.5	2.5	2.5	2.5			
Calculated analysis															
ME, kcal/kg	3024	3022	3022	3022	3100	3099	3099	3099	3151	3148	3148	3148			
Crude protein	22.3	22.2	22.2	22.2	20.6	20.6	20.6	20.6	19.2	19.2	19.2	19.2	6.0	5.6	5.7
Crude fiber	2.44	2.91	2.91	2.91	2.36	3.01	3.01	3.01	2.29	2.94	2.94	2.94	11.1	12.5	13.5
Ether extract	3.76	5.36	5.36	5.36	4.36	6.64	6.64	6.64	4.74	7.01	7.01	7.01	1.5	1.1	1.6
Ash	5.51	5.63	5.63	5.63	4.69	4.82	4.82	4.82	4.37	4.62	4.62	4.62	6.8	7.8	7.4
Calcium	1.00	1.02	1.02	1.02	0.81	0.83	0.83	0.83	0.77	0.78	0.78	0.78			
P (total)	0.83	0.82	0.82	0.82	0.73	0.72	0.72	0.72	0.67	0.67	0.67	0.67			
P (available)	0.53	0.52	0.52	0.52	0.44	0.44	0.44	0.44	0.40	0.40	0.40	0.40			
Lysine	1.46	1.47	1.47	1.47	1.29	1.30	1.30	1.30	1.15	1.16	1.16	1.16			
Methionine + cystine	1.10	1.10	1.10	1.10	1.01	1.02	1.02	1.02	1.15	1.16	1.16	1.16			

1 The vitamin and mineral premix provided per kg of diet: 10,000 IU of vitamin A (retinyl acetate); 5000 IU of cholecalciferol; 80 mg of vitamin E (DL-α-tocopheryl acetate); 8 mg of menadione; 3.5 mg of thiamine; 7.5 mg of riboflavin; 6.75 mg of pyridoxin; 50 µg of cobalamin; 62.5 mg of nicotinic acid; 25 mg of pantothenic acid; 2 mg of folic acid; 200 µg of biotin,; 8.5 mg of vitamin C (ascorbic acid); 500 mg of choline chloride; 1.2 mg of I; 0.35 mg of Se; 50 mg of Fe; 140 mg of Mn; 25 mg of Cu; and 115 mg of Zn.

2 Analyzed composition OP= orange pulp, T0=orange pulp in presence of mixed inoculum without fermentation, T20=orange pulp, fermented with mixed inoculant for 20 days,.

### Orange pulp fermentation

Orange pulp was obtained from a local orange juice industry (Hellenic Juice Industry C. Dedes ASPIS S.A., Argos, Greece) in a pelleted form. The inoculum consisted of orange juice, which was inoculated with indigenous microbiota that exhibited high pectinolytic, xylanolytic and cellulolytic activities and characterized by Zerva et al. (2019a,b; 2021), mainly consisted of members of the genera *Levilactobacillus* (ex. *Lactibacillus*) and *Saccharomyces,* to serve as the starter culture. Orange pulp pellets were milled and rehydrated by adding distilled water for the OP group (at ratio of 4:9 w/w orange pulp pellets: distilled water) and distilled water plus mixed inoculum for the T0 and T20 groups (at ratio of 4:6:3 w/w/w orange pulp pellets: distilled water: inoculum). Solid-state fermentation set-up (T20) was incubated at 27° C for a period of 20 days. Before their addition in the experimental diets, OP and T0 setups were followed by drying for 1 day at 50 °C, to 90 % dry matter, in order to avoid any form of fermentation whereas the T20 setup was dried at the end of the 20-day period of solid-state fermentation.

### Body weight, carcass and internal organ weights

At 42 days of age, eight chickens from each treatment group were randomly selected, individually weighed, electrically stunned, and slaughtered. The weights of carcass, gizzard, liver, bursa, spleen, heart and fat pad were recorded. Chicken carcasses were subsequently chilled at 4°C for 24 hours to facilitate the meat quality and oxidative stability assessment on the *pectoralis major* muscle.

### pH24 and color

pH24 was measured by inserting a pH meter probe (HI 99163 model, Hanna instruments, Nușfalău, Romania), into the right *pectoralis major* muscle 24 hours after slaughter. Meat color was assessed using three measurements per sample on the right *pectoralis major* muscle after a 30-min exposure to air at room temperature. Skin color was determined thrice at the skin of the thigh. Color was also measured in fermented and non-fermented OP feed samples. A Miniscan XE chromameter (HunterLab, Reston, VA) was used, operating on the L* (lightness), a* (redness), and b* (yellowness) color system, using illuminant D65 with 0° viewing. Calibration was performed using white and black tiles. Shank color was also determined with a DSM BroilerFan^TM^.

### Cooking loss and shear force value

The left *pectoralis major* muscle was dissected, weighed, and placed into a thin-walled plastic bag, then cooked in a water bath at 80°C for 30 min. After cooking, the muscle was cooled under tap water and allowed to equilibrate to room temperature. The muscle was weighed again to determine cooking loss (%). Shear force was assessed following the method described by Cason et al. (1997). Specifically, two strips (19 mm wide × 10 mm × 10 mm) were cut from the center of the muscle, parallel to the muscle fibers. The strips were then sheared perpendicular to the fiber direction using a Zwick Testing Machine Model z2.5/TN1S (Zwick GmbH & Co., Ulm, Germany) armed with a Warner-Bratzler shear blade. Warner–Bratzler shear blade specifications included a crosshead speed set at 200–250 mm/min, v-shaped (60° angle) cutting blade with a thickness of 1.016 mm, cutting edge beveled to a half-round, corner of the v rounded to a quarter-round of a 2.363 mm diameter circle and 2.032 mm-thick spacers providing the gap for the cutting blade to slide through. Peak force values were recorded in N/cm^2^.

### Intramuscular fat

Intramuscular fat content was determined following the method of Folch et al. (1957) in two samples per chicken. *Pectoralis major* muscle samples were mixed with a 2:1 chloroform-methanol solution (vol/vol) to achieve a final solvent volume 20 times greater than the tissue volume. The resulting extract was mixed with distilled water in a 5:1 ratio, leading to 2 phase separation. The lower phase, which contained the lipid fraction, was then collected for analysis.

### Oxidative stability

Lipid oxidation was assessed by measuring the secondary oxidation product malondialdehyde (MDA), released from the hydrolysis of lipid hydroperoxides. The MDA concentration in *pectoralis major* muscle samples (right side) was determined after slaughter and storage at 4°C for 1, 3, 6, and 9 days as well as at 20° C for 60 and 120 days using the third-order derivative spectrophotometric method developed by Botsoglou et al. (1994). Briefly, 2 g of each sample (two samples per chicken) were homogenized with 8 mL of aqueous trichloroacetic acid (50 g/L) and 5 mL of butylated hydroxytoluene in hexane (8 g/L). The mixture was centrifuged at 3,000 × *g* for 5 min at 4°C. The upper hexane layer was discarded, while 2.5 mL from the lower aqueous phase were mixed with 1.5 mL of aqueous 2-thiobarbituric acid (TBA; 8 g/L), followed by incubation at 70°C for 30 min. After cooling with tap water, MDA concentration (reported as ng/g wet tissue) was calculated from the height of the third-order derivative peak at 521.5 nm using a third-order derivative spectrophotometry (Hitachi U3010 Spectrophotometer, Tokyo, Japan) against a standard calibration curve made with the MDA precursor 1,1,3,3-tetraethoxypropane.

### Blood sampling, serum cholesterol

Two blood samples were collected from the chicks during slaughter. A 0.2 mL blood sample was used for the analysis of peripheral blood leukocytes as described below, and an additional 2 mL sample was collected into tubes and allowed to clot at room temperature for 15 min prior to centrifugation at 1,000 × *g* for 15 minutes. The supernatant serum was then obtained for the determination of cholesterol level. By employing a commercial cholesterol reagent kit (Biosis Commercial Kits; Athens, Greece), both serum total cholesterol and high-density lipoprotein (HDL) levels were determined at 540 nm in a Hitachi U3010 spectrophotometer.

### Flow cytometric peripheral blood leukocyte analysis

**Fig. 1:**
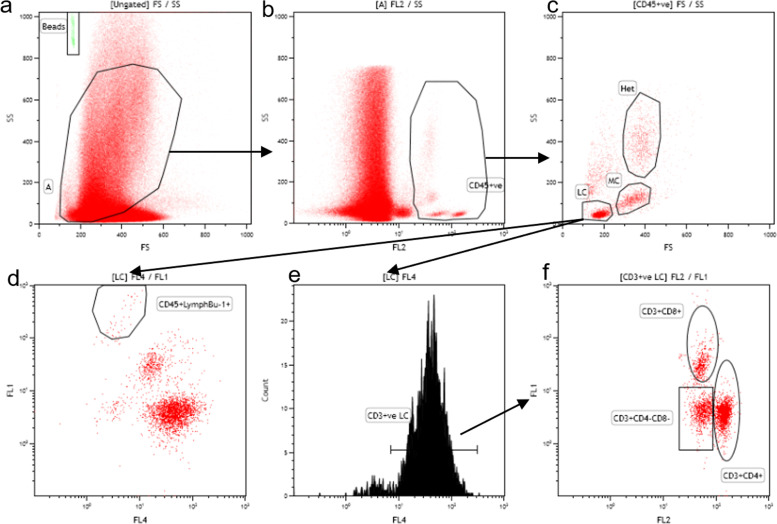
Identification of different peripheral blood leukocyte types by flow cytometry. (a). Forward/side scatter (FS/SS) dot plot of all events and region A is drawn based on expected leukocyte size and granularity. (b). Events in region A are plotted on a SS/FL2 (CD45) dot plot and cells positive for CD45 (CD45+ve) are identified as leukocytes. (c). Cells identified as leukocytes are plotted on an FS/SS dot plot and regions LC, MC and Het are used to identify lymphocytes, monocytes and polymorphonuclear granulocytes (mainly heterophils) respectively. (d). Lymphocytes are plotted on a FL1 (Bu-1)/FL4(CD3) dot plot and cells staining positively for Bu-1 are identified as B cells. Bu-1 and CD8 positive cells are both detected in the FL1 channel, as both anti-Bu-1 and anti-CD8 antibodies are conjugated with FITC fluorophore. Nevertheless, B cells are negative for CD3 (FL4) and fluorescence levels in FL1 is higher compared to CD8+ cells, that are positive for both FL1 and FL4 (population in the middle of the plot), thus allowing the clear distinction between the two lymphocyte types. (e). Lymphocytes are also plotted on a histogram for CD3 cell surface expression and positively stained cells are identified as T cells. (f). T cells are further plotted on a FL1 (CD8) / FL2(CD4) dot plot to identify T helper (Th, CD3+CD4+ve) and T cytotoxic (Tc, CD3+CD8+ve) cells. CD4+ *T* cells are detected in the same channel as all CD45+ cells (FL2) since anti-CD45 and anti-CD4 antibodies are both conjugated with PE-Cy5. Nevertheless, CD4+ *T* cells can be clearly distinguished from other leukocytes, as the level of fluorescence in FL2 is higher in the former.

Peripheral blood leukocytes (PBL) were analyzed by flow cytometry using a single step technique (Seliger et al., 2012). Initially, 20μl whole blood, which was collected with EDTA as the anticoagulant, was diluted in 980μl PBS. To stain with cell surface antibodies, 50μl of diluted blood samples were mixed with 20μl staining solution containing fluorophore conjugated antibodies (Abcam, Cambridge, UK), 0.1 μg mouse anti-Bu-1(AV20)-fluorescein isothiocyanate (FITC, Invitrogen, Frederick, Maryland, USA), 0.25 μg mouse anti-CD8-fluorescein isothiocyanate (FITC, Abcam, Cambridge, UK), 0.05 μg mouse anti-CD45(LT40)-phycoerythrin (PE, Invitrogen, Frederick, Maryland, USA), 0.05 μg mouse anti-CD4(CT-4)-phycoerythrin (PE, Abcam, Cambridge, UK), and 0.1 μg mouse anti-CD3(CT-3)-PE/Cyanine 5 (PE-Cy5, Abcam, Cambridge, UK) in staining buffer (PBS containing 0.01 % NaAzide and 0.2 % BSA) and incubated at room temperature for 45 min, in the dark. The staining cell suspension was mixed with 400μl of staining buffer containing flow check beads (FLOW CHECK PRO, Beckman Coulter Inc., USA) and analyzed on a Cytomics FC 500 flow cytometer (Beckman Coulter Inc., USA). All pipetting was performed with reverse pipetting to optimize volumetric accuracy and allow for absolute quantification of leukocytes. Instrument voltage/gain for detectors FS (Linear), SS (Linear), FL1 (Log, FITC detection), FL2 (Log, PE detection) and FL4 (Log, PE-Cy5 detection) were set at 600/2.0, 695/20.0, 580/1.0, 580/1.0, 580/1.0, respectively. Samples were run at maximum speed for 180 seconds and data was stored as list mode files and analyzed with Kaluza Analysis software version 1.3 (Beckman Coulter Inc., USA). For each sample, the remaining volume after flow cytometry analysis was estimated by pipetting and volume run was estimated by subtracting remaining volume from 470μl total sample volume. A correction factor for volume run (CF) was estimated as the average of BeadCount/VolumeRun for each sample, where BeadCount is the number of beads counted. Absolute cell counts for each cell type were estimated using the formula: AbsCellCount=[CellCount/(BeadCount/CF)]*470×1000 cells/ml, where CellCount is the respective number of cells counted in 180 sec run for each cell type, and BeadCount/CF is the corrected sample volume run, based on the number of beads counted (BeadCount). PBL cell types were identified as shown in Fig. 1.

### Statistical analysis

Experimental data were subjected to analysis of variance with the MIXED procedure of the SAS software (SAS Institute Inc., 2005). The fixed effect was the dietary treatment with fermented and non-fermented OP. Malondialdehyde data were analyzed with the MIXED procedure for repeated measures because measurements were repeatedly applied to the same breast meat sample at storage days 1, 3, 6, 9, 60 and 120. Dietary treatment, storage duration and their interaction were modeled as fixed effects, with storage duration being the repeated factor. Multiple comparisons were made after Bonferroni adjustment. Statistical significance was set at *P* = 0.05, while probability values of 0.05<*P* < 0.1 were considered as tendencies to differences. Results are presented as least squares means ± standard error of means (SEM).

## Results

Table 2 shows the results on the performance, carcass and internal organ weights of broiler chickens as affected by dietary supplementation with OP that was either fermented or not. Mean BW at slaughter was determined at 2933, 2996, 2818 and 2844 g for broilers of the C, OP, T0 and T20 treatment groups, respectively, with no significant differences detected among groups (*P* > 0.05). No effect of dietary OP, fermented or not, was also determined for FCR that ranged from 1.55 to 1.60 (*P* > 0.05). FI and carcass traits expressed either in grams or as percentages to BW, were not affected by dietary interventions (*P* > 0.05).

**Table 2:** Effect of dietary orange pulp, fermented through solid state fermentation, on broiler performance, carcass and internal organ weights at 42 days of the study.

			Treatment			
Parameter	C	OP	T0	T20	SEM	P-value
BW, g	2933	2996	2818	2844	138	0.785
Cumulative FI, g	4510	4649	4600	4479	130	0.779
FCR, g g^−1^	1.60	1.55	1.59	1.58	0.05	0.918
Carcass weight, g	2291	2339	2173	2213	111	0.715
DP, %	78.1	78.0	77.1	77.8	0.55	0.550
Liver, g	50.6	52.0	51.8	48.2	2.74	0.759
Liver, %	1.73	1.74	1.83	1.70	0.06	0.498
Heart, g	11.6	12.7	13.0	11.3	0.89	0.480
Heart, %	0.40	0.42	0.46	0.40	0.03	0.213
Gizzard, g	36.4	37.3	36.8	37.8	1.97	0.963
Gizzard, %	1.25	1.26	1.33	1.32	0.07	0.836
Fat pad, g	28.9	24.9	20.6	27.9	3.05	0.244
Fat pad, %	0.99	0.84	0.74	0.98	0.10	0.306
Spleen, g	2.25	2.29	2.63	2.28	0.16	0.300
Spleen, ‰	0.78	0.80	0.95	0.79	0.07	0.337
Bursa, g	1.33	1.57	1.59	1.51	0.20	0.804
Bursa, ‰	0.46	0.52	0.56	0.53	0.06	0.711
Thigh skin L*	61.2	60.3	60.3	60.8	0.83	0.069
Thigh skin a*	6.35^b^	7.85^a^	8.36^a^	6.88^ab^	0.42	0.009
Thigh skin b*	13.7	14.0	14.4	12.3	0.80	0.304
Shank colour DSM BroilerFan^TM^	104.0^b^	105.9^a^	105.3^a^	104.3^b^	0.21	<0.001

*C*= no additive, OP= orange pulp at 70 g per kg feed, T0= orange pulp in presence of mixed inoculum without fermentation, at 70 g per kg feed T20=orange pulp, fermented with mixed inoculum for 20 days, at 70 g per kg feed.

BW= body weight, FI= feed intake, FCR= Feed conversion ratio, DP= Dressing percentage, %, ‰ of BW.

L*=lightness, a*=redness, b*=yellowness.

*n* = 8, except FI and FCR where *n* = 4 replicate pens.

a,b Means in the same row with different superscripts are statistically different (*P* < 0.05).

On the other hand, color attributes of carcass were altered because of OP consumption by broilers. Skin color that was measured at thighs of broilers fed with OP, regardless of fermentation, tended to be darker in comparison with controls as shown from the tendency for reduced L values in OP, T0 and T20 groups (*P* < 0.1). The increased a* color parameter determined for the OP and T0 groups, 7.85 and 8.36, respectively, in comparison with 6.35 for C group (*P* < 0.05) are indicative of a more reddish skin color of carcasses obtained from chickens fed with non-fermented OP in comparison with those fed with a control diet. Skin color parameter a* of broilers fed with fermented orange pulp (FOP) was determined at 6.88 and did not differ from that of the C group nor from that of the OP or T0 groups (*P* > 0.05). The skin coloring effect of dietary non-fermented OP was more pronounced when color was measured at the shank with DSM BroilerFan^TM^. Shanks of broilers fed with non-fermented OP showed increased DSM BroilerFan values (105.9 and 105.3 for OP and T0 groups), respectively, in comparison to C and T20 groups (104.0 and 104.3, respectively, *P* < 0.05). The influence of OP on skin color was also reflected on meat color that was measured on the *pectoralis major* muscle (Table 3). Reduced a* color parameter for redness were obtained from the breast meat of broilers fed with fermented OP (4.94) in comparison with those from the non-fermented OP supplemented groups OP and T0 (6.85 and 6.55, respectively, *P* < 0.05). These differences are indicative of a more reddish color for meat obtained from broilers fed with non-fermented OP in comparison to those fed with fermented OP. The rest of the meat quality traits measured, pH_24_, breast intramuscular fat, cooking loss, and shear force were not affected by dietary treatment (*P* > 0.05). Because skin color is usually affected by feed pigments, color of all form of OP fed to animals was measured. Color attributes for non-fermented OP, OP inoculated with mixed inoculum but not fermented, and orange pulp fermented for 20 days were determined at 58.0, 55.9 and 58.2 for the L* parameter, 9.45, 10.8 and 8.1 for the a* parameter and 34.2, 39.1 and 29.6 for the b* parameter, respectively. As shown, fermented OP showed reduced redness and yellowness in comparison with non-fermented OP.

**Table 3:** Effect of dietary orange pulp, fermented through solid state fermentation, on broiler meat quality and serum cholesterol.

			Treatment			
Parameter	C	OP	T0	T20	SEM	P-value
pH_24_	6.00	6.05	6.04	6.06	0.05	0.837
Breast intramuscular fat, %	1.55	1.27	1.70	1.30	0.14	0.115
Cooking loss, %	11.9	10.1	11.4	10.3	0.59	0.102
Shear force, N/cm^2^	33.1	36.0	32.9	40.3	2.91	0.258
Breast meat L*	60.4	58.6	57.5	58.8	0.95	0.212
Breast meat a*	5.58^ab^	6.85^a^	6.55^a^	4.94^b^	0.33	0.001
Breast meat b*	17.7	17.3	17.4	17.0	0.66	0.897
Serum cholesterol, mg/dl	119.7^a^	95.6^b^	106.2^ab^	112.3^ab^	4.18	0.003
Serum HDL, mg/dl	75.4	54.8	60.8	58.7	5.50	0.079

*C*= no additive, OP= orange pulp at 70 g per kg feed, T0= orange pulp in presence of mixed inoculum without fermentation, at 70 g per kg feed T20=orange pulp, fermented with mixed inoculum for 20 days, at 70 g per kg feed.

L*=lightness, a*=redness, b*=yellowness.

HDL= high density lipoprotein.

*n* = 8.

a,b Means in a row sharing no common superscript are statistically different (*P* < 0.05).

Results of serum total and HDL cholesterol levels are presented in Table 3. Total cholesterol was determined significantly lower at 95.6 mg/dl in the OP group in comparison with C group where it was determined at 119.7 mg/dl (*P* < 0.05) with intermediate levels determined for the T0 and T20 groups. A tendency for reduced serum HDL cholesterol levels was detected for OP supplemented groups, regardless of fermentation, 54.8, 60.8 and 58.7 mg/dl for OP, T0 and T20 groups, respectively, in comparison with 75.4 mg/dl for C group (*P* < 0.1).

Meat oxidative stability results are presented in (Table 4). Oxidative stability throughout refrigerated storage, for 9 days at 4° C, expressed in ng of MDA per g of breast meat, was found to be improved in group of birds fed with FOP (T20) (9.01 ng/g) and the basal diet (7.94 ng/g) in comparison with those fed with non-fermented orange pulp (17.4 and 18.6 ng/g, for OP and T0 groups, respectively, *P* < 0.05). Significant differences were detected at 1, 6 and 9 days of storage (*P* < 0.05). The same pattern was observed when meat samples were stored in the freezer for 60 and 120 days, with lower MDA values determined for C and T20 groups in comparison with non-fermented OP supplemented groups. However, significant differences were detected only between control (7.90 ng/g) and the OP group (12.1 ng/g, *P* < 0.05).

**Table 4:** Effect of dietary orange pulp, fermented through solid state fermentation, on oxidative stability of broiler breast meat during storage for 1 to 9 days at 4°, and for 60 to 120 days at −20° C (ng of malondialdehyde/g of breast meat).

		Treatment				
Storage time, days	C	OP	T0	T20	SEM	P-value
1-9*	7.94^b^	17.4^a^	18.6^a^	9.01^b^	1.97	<0.001
1	3.77^b^	4.87^a^	4.62^a^	4.24^ab^	0.22	0.008
3	4.71	6.75	6.03	5.09	0.71	0.187
6	9.47^b^	22.7^a^	26.8^a^	11.0^b^	2.98	<0.001
9	13.8^b^	35.4^a^	36.8^a^	15.7^b^	4.61	0.001
60-120*	7.90^b^	12.1^a^	11.3^ab^	8.80^ab^	1.00	0.018
60	7.46	10.3	9.51	7.84	1.07	0.221
90	8.33^b^	14.0^a^	13.0^ab^	9.77^ab^	1.19	0.007

*C*= no additive, OP= orange pulp at 70 g per kg feed, T0= orange pulp in presence of mixed inoculum without fermentation, at 70 g per kg feed T20=orange pulp, fermented with mixed inoculum for 20 days, at 70 g per kg feed.

*Least square means of the repeated measures analysis for the entire storage period.

*n* = 8.

a,b Means in a row sharing no common superscript are statistically different (*P* < 0.05).

Results on measurements of different types of PBL determined by flow cytometry on broiler blood samples are presented in Table 5. No significant effect of OP supplementation, fermented or not, was observed in heterophils, lymphocytes (L), monocytes, B cells, T cells and T helper (Th) cells (*P* > 0.05). The only leukocyte parameter influenced by dietary intervention was the number of T cytotoxic cells (Tc) that were decreased in OP and T0 groups (0.96 and 0.95×10^6^, respectively) in comparison with Control (1.25×10^6^) and T20 group (1.08×10^6^), with significant differences detected when compared with controls (*P* < 0.05). A similar decrease in the % of T cytotoxic cells in non-fermented OP groups was observed, but differences were not statistically significant (*P* = 0.111). The H/L and Th/Tc ratios were not affected by dietary intervention with fermented and non-fermented OP (*P* > 0.05).

**Table 5:** Effect of dietary orange pulp, fermented through solid state fermentation, on cellular immunity parameters of broiler chickens.

		Treatment						
Parameter	C	OP	T0	T20	SEM		P-value	
Leucocytes, x10^6^	19.47	18.84	17.38	17.83		1.20		0.608
Heterophils, x10^6^	3.57	3.61	3.18	3.46		0.31		0.742
Heterophils, %	25.8	26.8	26.8	27.5		1.76		0.928
Lymphocytes, x10^6^	7.71	6.69	6.36	6.20		0.62		0.325
Lymphocytes, %	55.2	50.3	51.8	49.2		2.27		0.282
Monocytes, x10^6^	2.62	3.10	2.58	2.90		0.26		0.483
Monocytes, %	18.9	22.9	21.4	23.3		1.41		0.140
B cells, x10^4^	10.0	5.54	5.75	8.27		1.65		0.191
B cells, %	0.80	0.41	0.49	0.65		0.15		0.284
T cells, x10^6^	7.04	6.22	5.89	5.71		0.50		0.274
T cells, %	51.0	46.8	48.2	45.3		2.33		0.376
T helper cells, x10^6^	3.50	3.16	2.98	2.86		0.28		0.411
T helper cells, %	25.6	24.2	24.2	22.7		1.89		0.764
T cytotoxic cells, x10^6^	1.25^a^	0.96^b^	0.95^b^	1.08^ab^		0.08		0.025
T cytotoxic cells, %	9.26	7.30	7.91	8.68		0.58		0.111
H/L ratio	0.48	0.54	0.53	0.59		0.06		0.655
Th/Tc ratio	2.79	3.34	3.15	2.72		0.27		0.313

*C*= no additive, OP= orange pulp at 70 g per kg feed, T0= orange pulp in presence of mixed inoculum without fermentation, at 70 g per kg feed T20=orange pulp, fermented with mixed inoculum for 20 days, at 70 g per kg feed.

*H*=Heterophils, *L*=Lymphocytes, Th=*T* helper cells, Tc= *T* cytotoxic cells, % percentage of total leukocytes.

*n* = 8.

a,b Means in the same row with different superscripts are statistically different (*P* < 0.05).

## Discussion

In this study, the effects of OP inclusion in broiler diets, either unfermented or subjected to solid-state fermentation for 20 days, on performance, carcass characteristics, meat quality, oxidative stability, blood lipid profiles and immune parameters, were investigated. No significant differences were identified in final body weight, feed intake, and feed conversion ratio among dietary treatments indicating that OP can be included in broiler diets at levels up to 7 % without negatively affecting growth performance. The number of observations for FI and FCR in our study (*n* = 4) are considered low for detecting subtle or small differences that actually exist. However, the large P-values that were determined, 0.779 for FI and 0.918 for FCR, suggest that an increased number of pens probably wouldn’t change results obtained. In agreement with our results, Yi et al. (2023) did not detect any effect of citrus pulp, fed at 1.5 and 2.5 %, on broiler performance. On the other hand, Murao et al. (2008) and Chaudry et al. (2004) reported broiler growth rate impairment when they were fed diets containing 10 % citrus pulp. Murao et al. (2008) attributed the negative effects on the increased levels of dietary non-starch polysaccharides in citrus pulp. The absence of negative effects on performance in the present study suggests that the fiber content of OP at the tested inclusion level did not exceed the digestive tolerance threshold of broilers, which is crucial since excessive fiber can limit nutrient digestibility and feed efficiency (Choct, 1997; Hetland and Svihus, 2001).

Interestingly, significant changes were detected in skin and meat color attributes. Broilers consuming non-fermented OP exhibited higher a* values, indicating redder skin and breast muscle. Citrus by-products are rich in natural pigments, including carotenoids such as β-cryptoxanthin and lutein, which can deposit in skin and muscle tissues, influencing poultry product appearance (Miao et al., 2024). The observed reduction in skin and meat redness in fermented OP-fed broilers suggests that fermentation might degrade or modify these pigment compounds (Yang et al., 2023; Yuan et al., 2024) and this is evidenced in the present study by the reduced redness of the fermented OP determined with the chromameter. The a* value of the fermented OP was detected lower than the values obtained for the non-fermented OP. This is consistent with findings indicating that microbial metabolism during fermentation can alter the profile of bioactive compounds, including pigments (Moukamnerd et al., 2025).

Serum total cholesterol was beneficially reduced in all OP-supplemented groups, regardless of fermentation. Citrus by-products are known for their high soluble fiber content, mainly pectin, and the presence of bioactive compounds, such as flavonoids like hesperidin, can modulate lipid metabolism by binding bile acids, reducing cholesterol absorption, or inhibiting hepatic cholesterol synthesis in rats (Bok et al., 1999) and humans (Gupta et al., 2025). These hypocholesterolemic effects suggest that utilization of citrus by-products in poultry feeding may promote the production of healthier food from poultry. However, the tendency for lower HDL cholesterol levels observed in the OP groups requires further investigation, as reduction in HDL may not be desirable from a cardiovascular health perspective (Fernandes et al., 2007). The hypocholesterolemic effects of OP observed in the present study are in line with those of Behera et al. (2019) who reported lower plasma cholesterol and triglyceride levels on broilers fed with citrus waste at 2.5, 5 and 7.5 % and Latif et al. (2023) who detected increased HDL levels in broilers fed with orange peel meal at 80, 160 and 240 g/kg of feed.

From a meat quality perspective, our most important finding was the improved oxidative stability of breast meat from broilers fed with fermented OP in comparison with those fed with unfermented OP, as indicated by significantly lower MDA concentrations during refrigerated storage in comparison with breast meat from broilers fed with unfermented OP. Surprisingly, the MDA concentration was detected much higher in meat samples from treatment groups fed with unfermented OP in comparison with either control or T20 groups. This difference may be explained by the increased fat content, by almost 50 %, of the experimental diets in comparison to control (Table 1) as a result of adding an excessive amount of soybean oil in the experimental diets that contained OP, almost twice than that in the control diet, in order to attain isoenergetic diets. This was because the addition of fermented and non-fermented OP in diets was applied at the expense of maize which is a high-energy feed source. The high unsaturated fatty acids content of soybean oil makes it prone to oxidation, which can lead to the formation of free radicals, a fact that increases rancidity which in turn may decrease meat oxidative stability when it is supplemented in animals’ diet (Amaral et al., 2018). In a similar manner this may explain the reduced oxidative stability detected in the OP and the T0 groups. The elevated content of polyunsaturated fatty acids makes poultry meat vulnerable to lipid oxidation, affecting its flavor, nutritional value and shelf-life (Estevez, 2015). The improved oxidative stability of the meat from broilers fed with fermented OP, and a diet with a fat content similar to the OP and T0 groups, suggests that fermentation enhanced the antioxidant capacity of OP, probably due to the increased release of phenolic compounds and the production of bioactive metabolites with radical-scavenging activity (Hur et al., 2014). This finding is particularly important in the context of reducing synthetic antioxidants in poultry meat production, in accordance with consumer preferences for natural additives (Botsoglou et al., 1994).

Dietary supplementation with orange pulp, regardless of fermentation, contrary to the benefits for meat quality and blood cholesterol levels, had minimal effects on the blood leukocyte profile. Heterophil, lymphocyte, monocyte, B cell, and T cell populations, were not altered by dietary treatment suggesting that under ordinary physiological conditions, the inclusion of OP at 70 g/kg feed does not cause a remarkable immune response. However, an exception was observed in the T cytotoxic (Tc) cell number, which was significantly lower in birds fed the diets with unfermented OP (OP and T0 groups) compared to the control group. The observed decrease may be indicative of a subtle immunosuppressive effect associated with the increased MDA values and consequently increased oxidative stress observed in treatment groups fed with non-fermented OP. Free radicals are associated with cell apoptosis, a set of morphological and biochemical changes occurring when cells undergo programmed cell death for maintaining normal cellular homeostasis (Redza-Dutordoir et al., 2016). However, excessive free radicals’ production during oxidative stress can impair T cell function and induce apoptosis in cytotoxic T and natural killer cells affecting the overall immune process (Thoren et al., 2006; Chandimali et al., 2025). Interestingly, birds fed the fermented pulp (T20) did not show a significant reduction in Tc cells compared to control, suggesting that the fermentation process can mitigate oxidative stress and thus protect the birds from the potential negative effects on immune cell populations. The fact that the H/L ratio and the Th/Tc ratios were unaffected by OP dietary supplementation further supports our conclusion that OP supplementation does not except a notable effect on broiler immune status. These indices are often used as stress indicators in poultry (Maxwell, 1993) and their constancy among treatments indicates that dietary OP did not cause a physiological challenge. On the other hand, Pourhossein et al. (2015) reported improved humoral immune response in broilers fed with a sweet orange peel extract, in terms of an increased antibody titer against Newcastle and infectious bursal diseases and a beneficially reduced H/L ratio. The different form of orange pulp fed to animals that may have resulted in different concentration in bioactive compounds may probably explain differences between the two studies.

In conclusion, this study showed that orange pulp, an abundant by-product of the citrus industry, can be successfully utilized in broiler feeding, at 70g/kg feed, without negative effects on growth performance or carcass traits. When broilers are fed with diets with increased fat content, fermentation of OP for 20 days enhances meat oxidative stability in comparison with unfermented OP and alleviates the negative effects of oxidative stress on parameters of the immune system. Feeding broilers with OP partially modified skin and meat pigmentation and reduced serum cholesterol levels. Indeed, introducing fermented OP in broiler nutrition is in line with fundamentals of the circular economy and contributes to sustainable poultry farming through the valorization of these abundant agro-industrial residues. Further research is required to elucidate the key bioactive compounds responsible for the beneficial effects observed, and the economic viability of application of OP fermentation in commercial scale.

## CRediT authorship contribution statement

**Michael Goliomytis:** Writing – original draft, Supervision, Resources, Project administration, Methodology, Investigation, Funding acquisition, Data curation, Conceptualization. **Panagiotis Simitzis:** Writing – review & editing, Methodology, Investigation. **Agori Karageorgou:** Writing – review & editing, Methodology, Investigation. **Nicoleta Michalea:** Writing – review & editing, Methodology, Investigation. **Kyriaki Belesi:** Writing – review & editing, Methodology, Investigation. **Maria-Eleni Mougiou:** Writing – review & editing, Methodology, Investigation. **Vasiliki Syritou:** Writing – review & editing, Methodology, Investigation. **Ariadne-Loukia Hager-Theodorides:** Writing – review & editing, Methodology, Investigation. **Ioannis Stavrakakis:** Writing – review & editing, Methodology, Investigation. **Spyridon Ntougias:** Writing – review & editing, Methodology, Investigation, Funding acquisition.

## Disclosures

Michael Goliomytis reports financial support was provided by Hellenic Foundation for Research and Innovation. If there are other authors, they declare that they have no known competing financial interests or personal relationships that could have appeared to influence the work reported in this paper.
